# Hypocalcemia Following Thyroidectomy in a Patient With COVID-19: A Case Report and Literature Review

**DOI:** 10.7759/cureus.66665

**Published:** 2024-08-12

**Authors:** Takahiro Inoue, Takumi Kumai, Kenzo Ohara, Miki Takahara

**Affiliations:** 1 Department of Otolaryngology - Head and Neck Surgery, Asahikawa Medical University, Asahikawa, JPN

**Keywords:** vitamine d, covid-19, parathyroid hormone, thyroidectomy, hypocalcemia

## Abstract

COVID-19 can lead to various complications, including severe respiratory symptoms. Both viral infections and total thyroidectomy are known to cause hypocalcemia, making a history of thyroidectomy a potential risk factor for hypocalcemia in COVID-19 patients. We present the case of a 34-year-old woman with Graves’ disease who developed hypocalcemia due to COVID-19 following a total thyroidectomy. The patient underwent an uneventful total thyroidectomy, with preservation of at least three of the four parathyroid glands. Postoperatively, her parathyroid hormone (PTH) levels were normal, and she was discharged without tetany. However, on postoperative day 90, she experienced mild hypocalcemia during a COVID-19 infection, although it was asymptomatic. By postoperative day 127, she presented with severe tetany and general malaise. Testing confirmed a reinfection with SARS-CoV-2 and hypocalcemia, while PTH levels remained normal. Treatment with intravenous calcium gluconate, oral calcium lactate, and alfacalcidol effectively resolved the hypocalcemia and tetany. The patient was subsequently discharged without tetany and has since been monitored without the need for calcium or vitamin D supplementation. This case highlights that the COVID-19 infection following a total thyroidectomy can cause hypocalcemia. Postoperative hypocalcemia is a common issue in head and neck surgery, and viral infections like COVID-19 should be considered in the differential diagnosis of hypocalcemia.

## Introduction

The outbreak of COVID-19, caused by SARS-CoV-2, has profoundly impacted global medicine. Despite the development of vaccines, many individuals continue to experience COVID-19, which can lead to various serious complications. Beyond respiratory issues, endocrine disturbances, particularly hypocalcemia, have been documented as complications of COVID-19 [1]. Additionally, total thyroidectomy is known to cause hypocalcemia, with reported incidence rates ranging from 2% to 83% [2]. Here, we present a case of hypocalcemia following a total thyroidectomy, attributed to COVID-19.

## Case presentation

A 34-year-old woman with Graves’ disease underwent medical treatment for a year at a private clinic. She was referred to our department for a total thyroidectomy due to inadequate control of thyroid function with thiamazole (20 mg/day) and potassium iodide (50 mg/day). The patient had a history of asthma and had not been vaccinated against SARS-CoV-2. At her initial visit to our department, the laboratory data were as follows: albumin: 4.5 g/dL; calcium: 9.2 mg/dL; phosphate: 3.4 mg/dL; free triiodothyronine: 2.58 pg/mL; free thyroxine: 0.78 ng/mL; thyroid-stimulating hormone: 14 μIU/mL; intact parathyroid hormone (PTH): 46.5 pg/mL; thyrotropin receptor antibody: 10.6 IU/L; anti-thyroglobulin antibody: 12.3 IU/mL; and anti-thyroid peroxidase antibody: 9 IU/mL (Table 1).

**Table 1:** Preoperative laboratory data

Parameter	Measured value	Normal range
Albumin	4.5 g/dL	4.1-5.1 g/dL
Calcium	9.2 mg/dL	8.8-10.1 mg/dL
Phosphate	3.4 mg/dL	2.7-4.6 mg/dL
Free triiodothyronine	2.58 pg/mL	2.30-4.00 pg/mL
Free thyroxine	0.78 ng/dL	0.90-1.7 ng/dL
Thyroid-stimulating hormone	14 μIU/mL	0.50-5.00 μIU/mL
Intact parathyroid hormone	46.5 pg/mL	15.00-65.00 pg/mL
Thyrotropin receptor antibody	10.6 IU/L	0.00-1.99 IU/L
Anti‐thyroglobulin antibody	12.3 IU/mL	<28 IU/mL
Anti-thyroid peroxidase antibody	9 IU/mL	<16 IU/mL

**Figure 1:**
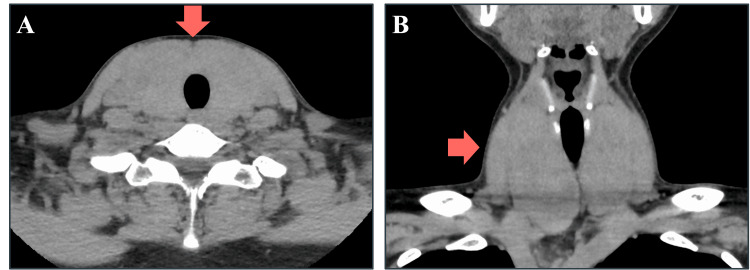
CT image of the thyroid The thyroid gland was enlarged to a maximum width of approximately 7 cm. (A) Axial CT scan showing a diffusely enlarged thyroid gland. (B) Coronal CT scan displaying the same diffuse enlargement of the thyroid gland.

CT revealed a diffusely enlarged thyroid gland with a maximum width of approximately 7 cm (Figure 1A, 1B).

**Figure 2:**
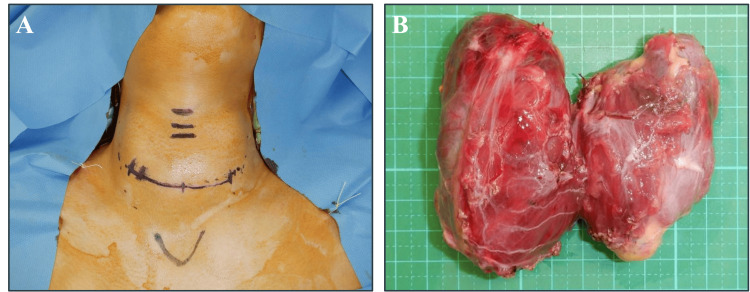
Intraoperative findings (A) Total thyroidectomy performed through a 7-cm transverse cervical incision. (B) Removed thyroid gland, weighing 125 grams.

We performed a total thyroidectomy through a transverse cervical incision under general anesthesia (Figure 2A). At least three out of four parathyroid glands were preserved, and the surgery was completed without complications. The excised thyroid gland weighed 125 g (Figure 2B). The patient received a calcium gluconate infusion for a few days post-surgery and was discharged with oral calcium lactate (3 g/day), alfacalcidol (2 μg/day), and levothyroxine. Follow-up blood tests showed normal levels of PTH and calcium without any signs of tetany, leading to the discontinuation of calcium lactate and alfacalcidol.

**Figure 3:**
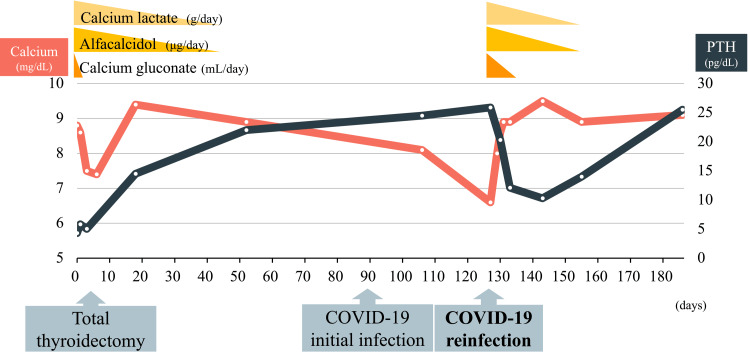
Postoperative course of corrected calcium and PTH levels The PTH level gradually increased following total thyroidectomy, with calcium supplementation concluding one month after surgery. Despite normal PTH levels, severe hypocalcemia occurred following the COVID-19 reinfection. Calcium administration resolved the symptoms of reactive PTH downregulation. After recovery from COVID-19, both calcium and PTH levels remained within the normal range without supplementation. PTH: parathyroid hormone

The patient experienced an initial SARS-CoV-2 infection on postoperative day 90, with mild symptoms that resolved spontaneously. Although there was a slight decrease in calcium levels to 8.1 mg/dL, the PTH level remained normal, and no tetany was observed (Figure 3).

**Figure 4:**
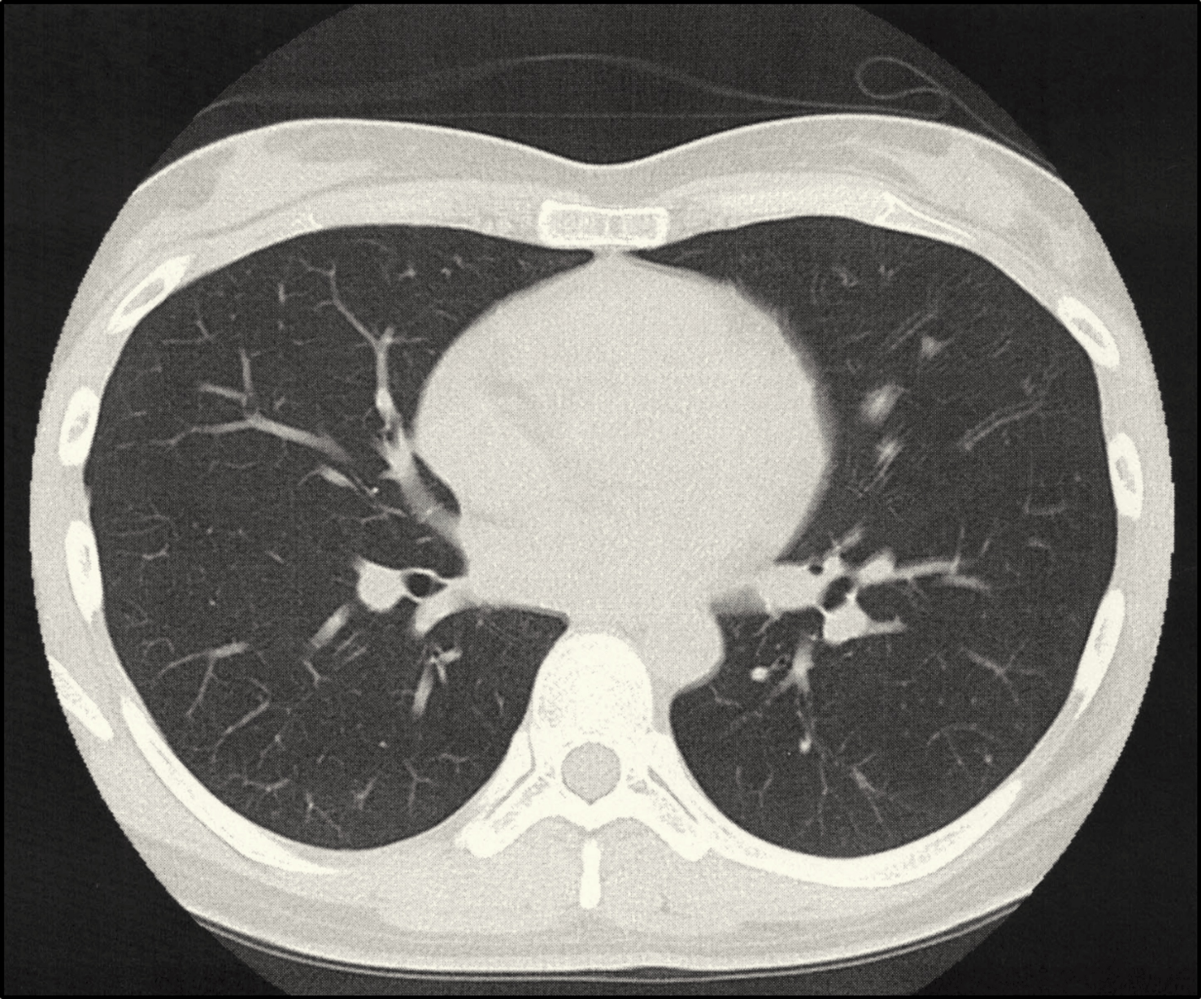
CT scan of the chest The chest CT scan revealed no evidence of pneumonia.

The patient was reinfected with SARS-CoV-2 on postoperative day 127, presenting with severe tetany, difficulty walking, general malaise, cough, and nasal discharge. Both SARS-CoV-2 antigen and PCR tests were positive, with a low PCR threshold cycle value of 23 indicating reinfection. A chest CT revealed no evidence of pneumonia (Figure 4).

The complete blood count was normal. Other laboratory results showed: albumin: 4.3 g/dL, calcium: 6.6 mg/dL, phosphate: 3.2 mg/dL, sodium: 138 mmol/L, potassium: 3.3 mmol/L, magnesium: 1.8 mg/dL, 1,25-(OH) vitamin D: 37 pg/mL, blood sugar: 110 mg/dL, blood urea nitrogen: 7.5 mg/dL, creatinine: 0.62 mg/dL, and PTH: 25.9 pg/mL (Table 2).

**Table 2:** Laboratory data during COVID-19 reinfection

Parameter	Measured value	Normal range
Albumin	4.3 g/dL	4.1-5.1 g/dL
Calcium	6.6 mg/dL	8.8-10.1 mg/dL
Phosphate	3.2 mg/dL	2.7-4.6 mg/dL
Free triiodothyronine	1.69 pg/mL	2.30-4.00 pg/mL
Free thyroxine	1.36 ng/dL	0.90-1.7 ng/dL
Thyroid-stimulating hormone	1.5 μIU/mL	0.50-5.00 μIU/mL
Intact parathyroid hormone	25.9 pg/mL	15.00-65.00 pg/mL
Sodium	138 mmol/L	138-145 mmol/L
Potassium	3.3 mmol/L	2.7-4.6 mg/dL
Magnesium	1.8 mg/dL	1.7-2.6 mg/dL
1.25-(OH) Vitamin D	37 pg/mL	20-60 pg/ml
Blood sugar	110 mg/dL	73-110 mg/dL
Blood urea nitrogen	7.5 mg/dL	8.0-20.0 mg/dL
Creatinine	0.62 mg/dL	0.46-0.79 mg/dL

Thyroid function remained normal with oral levothyroxine administration. Given the patient’s hypocalcemia with normal PTH levels, renal function, and vitamin D levels, COVID-19 was considered the cause of hypocalcemia. Hypocalcemia and tetany improved immediately with intravenous calcium gluconate, oral calcium lactate (3 g/day), and alfacalcidol (3 μg/day). Her malaise gradually resolved, and she was discharged 17 days after admission. The doses of calcium lactate and alfacalcidol were tapered off, and PTH levels, which had decreased in response to calcium supplementation, normalized. One year after the COVID-19 infection, the patient showed no signs of tetany and maintained normal calcium and PTH levels without medication.

## Discussion

Hypoparathyroidism, pseudohypoparathyroidism, vitamin D deficiency, and renal failure are the primary causes of hypocalcemia. Since Bossoni et al. reported the first case of severe hypocalcemia associated with COVID-19 in 2020 [3], the virus has been recognized as a potential inducer of hypocalcemia. The incidence of hypocalcemia among hospitalized COVID-19 patients ranges from 62.6% to 87.2% [4,5]. Given that hypocalcemia may serve as a prognostic marker for ventilator requirements and mortality in COVID-19 patients [6], normalization of calcium levels is crucial for effective management.

Virus-dependent calcium influx into cells, vitamin D deficiency, functional hypoparathyroidism, and PTH resistance are considered causes of hypocalcemia in COVID-19 [5]. Viruses utilize calcium signaling to enhance replication, with calcium playing a critical role throughout the viral life cycle, including viral structure formation, entry, gene expression, protein synthesis, fusion with host cells, and release of mature viruses [6]. In enveloped viruses such as SARS-CoV and Ebola, calcium directly interacts with viral fusion peptides to promote replication [7]. During the 2003 SARS and 2016 Ebola epidemics, hypocalcemia was observed in approximately 60% of affected patients [7]. Additionally, patients with COVID-19 often experience vitamin D deficiency, which can contribute to hypocalcemia [8]; however, vitamin D levels in our patient were normal. Functional hypoparathyroidism and PTH resistance, which have been reported in critically ill patients, may also be relevant in COVID-19 cases [9].

To date, 13 cases of COVID-19 with hypocalcemia have been reported, including the current case (Table 3) [3,10-20].

**Table 3:** Summary of reported cases of hypocalcemia associated with COVID-19 Calcium levels are adjusted by albumin (4 - albumin + calcium). PTH: parathyroid hormone

Case	Year	Author	Age/sex	Calcium (mg/dL)	PTH (pg/mL)	Phosphate (mg/dL)	Vitamin D (pg/mL)	Outcome of COVID-19	History
1	2020	Bossoni et al. [3]	72/F	4.75	10	5.2	8	Recovered	Total thyroidectomy
2	2020	Demir et al. [10]	68/F	6.2	2.8	7.8	5.3	Died	
3	2021	Bonnet et al. [11]	82/F	6.9	8.9	2.9	44	Recovered	
4	2021	Dianatfar et al. [12]	44/F	6.3	3	5.7	33	Recovered	
5	2021	Heidarpour et al. [13]	22/M	5	145	-	32	Died	
6	2021	Puca et al. [14]	81/F	5.7	107	-	4.5	Recovered	
7	2022	Azanjac et al. [15]	62/M	5	4.2	-	10	Died	
8	2022	Georgakopoulou et al. [16]	53/M	6.9	11.7	4.7	38.4	Recovered	
9	2022	Grigoravičius et al. [17]	39/M	4.4	3	6.5	15	Recovered	
10	2022	Irisson-Mora et al. [18]	63/F	4.8	2.1	8.3	31	Died	
11	2023	Bitew et al. [19]	48/M	2.6	12	11.2	7	Recovered	
12	2024	Selva et al. [20]	14/M	3.5	14.7	10	21	Recovered	
13	2024	Present case	34/F	6.6	25.9	3.2	37	Recovered	Total thyroidectomy

Of these patients, 46% (n = 6) were women. The median age was 53 years (range: 14-82 years). The median calcium level, adjusted for albumin, was 5.0 mg/dL (range: 2.6-6.9 mg/dL). Nine patients recovered, while four died from the infection. Notably, hypocalcemia with normal PTH levels (10-65 pg/mL) was observed in seven cases. It is possible that PTH levels were actually low but appeared normal due to the hypocalcemia. An additional six patients were diagnosed with hypoparathyroidism.

In Bossoni et al.’s case report, a patient who had undergone total thyroidectomy 19 years prior to the COVID-19 infection had normal calcium levels before the infection but developed hypocalcemia afterward [3], similar to our case. Our patient, who also underwent a total thyroidectomy, experienced hypocalcemia triggered by COVID-19. We observed that the initial COVID-19 infection caused mild hypocalcemia, which was exacerbated by a subsequent infection. Although most parathyroids were preserved and PTH levels seemed normal, it is plausible that parathyroid function was lower compared to patients who had not undergone total thyroidectomy. Hypocalcemia with normal PTH may be attributed to increased calcium demand due to virus-dependent calcium influx or PTH resistance.

A limitation of this study is the use of 1,25-(OH) vitamin D as a marker of vitamin D deficiency. While 1,25-(OH) vitamin D measures activated vitamin D, 25-(OH) vitamin D, which reflects the total amount of vitamin D, should also be assessed for a complete diagnosis of vitamin D deficiency.

Clinicians should be aware that a history of thyroid surgery combined with viral infections, including COVID-19, may increase the risk of hypocalcemia, even when PTH levels are normal. Further research is needed to determine whether residual parathyroid function after thyroidectomy is adequate to maintain serum calcium levels during high calcium demands, such as during viral infections.

## Conclusions

We report the case of a patient who developed hypocalcemia due to a COVID-19 infection following a total thyroidectomy. Despite normal vitamin D and PTH levels, COVID-19 was identified as the cause of hypocalcemia. While hypocalcemia is commonly observed after head and neck treatments, including total thyroidectomy, head and neck surgeons should be aware of viral infections as a potential contributing factor.
